# A comparison of generic drug prices in seven European countries: a methodological analysis

**DOI:** 10.1186/s12913-017-2184-5

**Published:** 2017-03-31

**Authors:** Olivier J. Wouters, Panos G. Kanavos

**Affiliations:** LSE Health, London School of Economics and Political Science, Houghton Street, London, WC2A 2AE UK

**Keywords:** Generic medicines, Generic drugs, Prices, Expenditures, Pharmaceutical policy

## Abstract

**Background:**

Policymakers and researchers frequently compare the prices of medicines between countries. Such comparisons often serve as barometers of how pricing and reimbursement policies are performing. The aim of this study was to examine methodological challenges to comparing generic drug prices.

**Methods:**

We calculated all commonly used price indices based on 2013 IMS Health data on sales of 3156 generic drugs in seven European countries.

**Results:**

There were large differences in generic drug prices between countries. However, the results varied depending on the choice of index, base country, unit of volume, method of currency conversion, and therapeutic category. The results also differed depending on whether one looked at the prices charged by manufacturers or those charged by pharmacists.

**Conclusions:**

Price indices are a useful statistical approach for comparing drug prices across countries, but researchers and policymakers should interpret price indices with caution given their limitations. Price-index results are highly sensitive to the choice of method and sample. More research is needed to determine the drivers of price differences between countries. The data suggest that some governments should aim to reduce distribution costs for generic drugs.

**Electronic supplementary material:**

The online version of this article (doi:10.1186/s12913-017-2184-5) contains supplementary material, which is available to authorized users.

## Background

Many European countries are facing severe cost pressures on health-care budgets, in part due to rising drug spending. In this context, the savings from greater use of generic drugs can help pay for other health-care services. Yet recent European Commission reports point to market failures for generic drugs [1, 2]. It is therefore important to regularly compare generic drug prices in countries with similar income levels in order to give public and private insurers a sense of whether they are over-paying for generic drugs or not. Such comparisons can serve as barometers of how pricing and reimbursement policies are performing [3–15].

Previous comparisons of generic drug prices have found that prices varied markedly across European and North American countries [16–24]. However, the studies often relied on different methods and samples, making it difficult to compare findings. In addition, most of the analyses had small sample sizes, which may have biased the results. Some earlier findings are also likely out of date given how often pricing and reimbursement regulations are changed.

As important, the impact of distribution margins and taxes on generic drug prices has been underexplored, even though studies indicate that those costs can account for more than 90% of the retail price of a generic drug, i.e., the price charged by pharmacists to patients or third-party payers [1]. Nearly all studies have looked at ex-manufacturer prices, i.e., those charged by manufacturers to wholesalers, which do not account for distribution costs.

In this study, we compared the ex-manufacturer and retail prices of a large sample of generic drugs in seven European countries. We calculated all commonly used price indices to outline the methodological challenges to comparing generic drug prices. It is critical that policymakers are aware of the advantages and limitations of these types of analyses, given that the results of price comparisons might be used to justify changes to pharmaceutical policies.

## Methods

We acquired 2013 data from IMS Health on volumes and sales of 200 off-patent active ingredients in seven countries with similar income levels: Belgium, Denmark, France, Germany, Italy, Spain, and Sweden. These ingredients were available in 3156 strength-form combinations.^1^ Volumes were recorded in doses and grams of active ingredient.^2^ Sales were recorded in euros based on average exchange rates for the year.^3^ We excluded 213 products (6.7%, 213/3156) with missing volume data.

We restricted our analysis to the 110 active ingredients sold in all seven countries, which accounted for 54 (Italy) to 87% (Sweden) of total generic spend in each country. For each ingredient, we calculated the average price per dose and the average price per gram, both at the ex-manufacturer and retail levels. To do this, we divided total sales in euros across strength-form combinations by number of doses or grams sold.^4^

We then calculated four indices — unweighted, Paasche, Laspeyres, and Fisher — using prices per gram and prices per dose [25]. Unweighted indices (I_U_) were calculated as

$$ {I}_U=\frac{{\displaystyle {\sum}_i}{p}_i^c}{{\displaystyle {\sum}_i}{p}_i^b} \cdot 100 $$

where *p w*as the price of active ingredient *i* in the comparator country or the base country. We selected Germany as the base country, which takes a value of 100 in all indices.

The other indices were weighted to account for consumption patterns. Paasche (I_P_) and Laspeyres indices (I_L_) were computed as

$$ {I}_P=\frac{{\displaystyle {\sum}_i}{p}_i^c{q}_i^c}{{\displaystyle {\sum}_i}{p}_i^b{q}_i^c} \cdot 100 $$

and

$$ {I}_L=\frac{{\displaystyle {\sum}_i}{p}_i^c{q}_i^b}{{\displaystyle {\sum}_i}{p}_i^b{q}_i^b} \cdot 100 $$

where *q* was the quantity in the comparator or base country (i.e., doses or grams). Finally, Fisher indices (I_F_) were calculated as

$$ {I}_F=\sqrt{I_P\cdot {I}_L} $$

### Sensitivity and subgroup analyses

The results of Laspeyres indices can vary depending on which country is selected as the base, since this determines which quantity weights are used. For instance, atorvastatin, a cholesterol-reducing drug, was only the 40^th^ most prescribed generic drug in Germany, in terms of number of doses sold, whereas it was one of the ten most prescribed generic drugs in three of the other countries. As a sensitivity analysis, we re-calculated all the price indices with France as the base country.

The results of price indices can also differ depending on whether exchange rates or purchasing power parities (PPPs) are used to convert monetary values to a common currency. Since exchange rates are sensitive to currency fluctuations, we re-calculated all of the indices based on PPP conversion factors. PPPs, which are measured in national currency units per US dollar, account for cross-country differences in the prices of goods and services. In this way, they equalize the purchasing power of different currencies.

Finally, we compared the prices of generic drugs in different therapeutic subgroups. To do this, we categorized the 110 active ingredients by anatomical main groups using the ATC/DDD system developed by the World Health Organization Collaborating Centre for Drug Statistics Methodology. Additional file 1: Appendix 1 shows the breakdown of active ingredients by group. We excluded ingredients that belonged to more than one group. For example, timolol is a beta blocker used to treat both high blood pressure (ATC group C) and glaucoma (ATC group S). We then compared the prices of the active ingredients belonging to the two largest groups in our sample: Cardiovascular system drugs (*n =* 25) and nervous system drugs (*n* = 29). The subgroup analysis used exchange-rate conversions and Germany as the base country.

The full results of the sensitivity and subgroup analyses can be found in Additional file 1: Appendices 2–4.

## Results

### Ex-manufacturer vs. retail prices

**Fig. 1:**
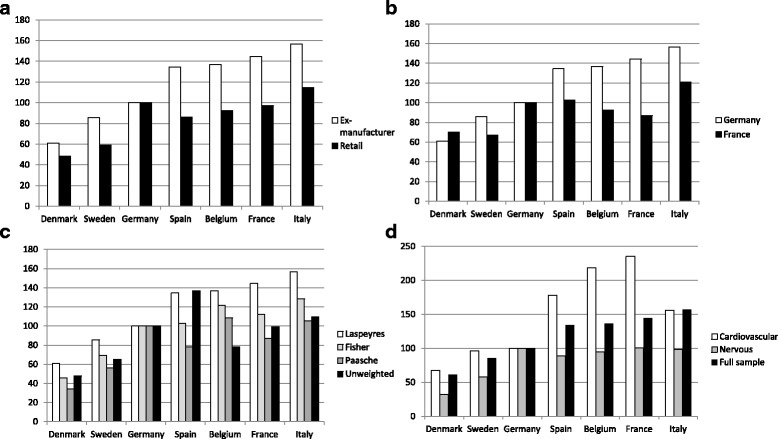
Results for different price indices in 2013 with Germany as the base country. For ease of interpretation, the unit of volume is doses in all the price indices. **a** Comparison of retail and ex-manufacturer prices (*n* = 110) in a Laspeyres index. **b** Contrast of ex-manufacturer prices (*n* = 110) in a Laspeyres index with German versus French weights. **c** Ex-manufacturer prices (*n* = 110) in weighted and unweighted indices. **d** Comparison of ex-manufacturer prices of cardiovascular system drugs (*n* = 25), nervous system drugs (*n* = 29), and all drugs (*n* = 110) in a Laspeyres index. (Source: IMS Health 2013, Pricing Insights database)

Table 1 summarizes the main results with Germany as the base country. Prices varied markedly across countries. Denmark and Sweden consistently had the lowest ex-manufacturer and retail prices among the seven countries, while France and Italy had the highest in most of the weighted indices. In the Laspeyres (dose) index, for example, the Italian ex-manufacturer prices were, on average, 1.6 times the German ones and 2.6 times the Danish ones. Figure 1a shows that while Belgium, France, and Spain all had higher ex-manufacturer prices than Germany, the opposite was true about their retail prices, based on a Laspeyres dose index.

**Table 1:** Ex-manufacturer and retail prices with Germany as the base country (2013)

	Belgium	Denmark	France	Germany	Italy	Spain	Sweden
Ex-manufacturer							
Unweighted-D	78.05	48.02	99.31	100.00	109.84	136.86	65.36
Unweighted-G	115.45	55.07	63.62	100.00	50.57	63.09	57.28
Laspeyres-D	136.67	61.11	144.46	100.00	156.66	134.47	85.70
Laspeyres-G	126.90	68.63	164.92	100.00	154.20	124.26	101.59
Paasche-D	108.75	34.38	87.07	100.00	105.59	78.32	56.50
Paasche-G	97.19	39.96	87.55	100.00	63.88	65.26	67.43
Fisher-D	121.92	45.84	112.15	100.00	128.62	102.62	69.58
Fisher-G	111.06	52.37	120.16	100.00	99.24	90.05	82.76
Retail							
Unweighted-D	70.97	48.96	98.38	100.00	117.57	129.61	52.91
Unweighted-G	104.09	58.36	57.03	100.00	56.97	62.75	47.49
Laspeyres-D	92.23	48.29	97.06	100.00	114.67	86.01	59.12
Laspeyres-G	87.29	54.93	114.85	100.00	112.28	79.22	69.87
Paasche-D	70.46	32.50	62.17	100.00	76.27	46.43	44.01
Paasche-G	63.99	37.28	62.92	100.00	47.73	40.55	52.69
Fisher-D	80.61	39.61	77.68	100.00	93.52	63.19	51.01
Fisher-G	74.73	45.25	85.01	100.00	73.20	56.68	60.68

*D* doses, *G* grams of active ingredient

Source: IMS Health 2013 (Pricing Insights database)

### Unit of volume (doses vs. grams of active ingredient)

The results of the unweighted indices fluctuated widely depending on which unit of volume was used (Table 1). By contrast, most of the weighted results remained similar across the two units of volume.^5^ There were some exceptions: In the Laspeyres indices, for example, the French ex-manufacturer prices were lower than those in Italy when doses were used, whereas they were higher when grams of active ingredients were used (Table 1).

### Weighting (Laspeyres vs. Paasche vs. Fisher)

The Paasche indices were always lower than the Laspeyres indices at both the ex-manufacturer and retail levels (Table 1 and Fig. 1b). The Fisher results — which are the geometric means of the Laspeyres and Paasche indices — fell between the latter two.

### Base country

Figure 1c shows that the Laspeyres values dropped in all countries, except Denmark, when the French weights were used.^6^ This indicates that those drugs which were more highly consumed in France than in Germany were also cheaper in most of the other countries.

### Currency conversion (exchange rates vs. purchasing power parities)

The results were largely unchanged when PPPs — rather than exchange rates — were used to convert sales in local currencies to a common unit. This suggests that variation in drug prices between these seven countries was, for the most part, not due to differences in the costs of goods and services.

### Subgroup analyses

Figure 1d shows the ex-manufacturer prices of cardiovascular system drugs and nervous system drugs. The amount of price variation differed across therapeutic groups. In the full sample, there was a 2.5-fold difference in prices between the countries with the highest and lowest prices. By comparison, there were 3.1 and 3.5-fold differences in the prices of nervous system and cardiovascular drugs, respectively. Germany had the second highest prices for nervous system drugs, whereas it had among the lowest prices for cardiovascular system drugs.

## Discussion

In this analysis, we explored differences in the ex-manufacturer and retail prices of generic drugs across seven countries in 2013 using various price indices.

The ex-manufacturer and retail prices varied widely across countries. This is consistent with earlier studies comparing the prices of patented drugs at both levels [1, 13, 14]. More research is needed to disentangle the impact of supply- and demand-side policies, such as pricing, reimbursement, prescribing, and substitution rules, on the ex-manufacturer and retail prices of generics [26]. Prices variation is also likely due, in part, to differences in the regulation of wholesaler and pharmacy margins [1].

There are various methods for comparing drug prices across settings [25, 27], and they often produce remarkably different results. For example, the ex-manufacturer Laspeyres index (dose) in Table 1 suggests that the sample of generic drugs was about 60% more expensive in Italy than in Germany. On the other hand, the ex-manufacturer Paasche index (grams of active ingredient) indicates that the sample was about 35% cheaper in Italy than in Germany.

There were even larger differences between some of the weighted and unweighted indices. It might be especially important to use weighted indices when comparing generic drug prices, since studies suggest that these prices are closely linked to volume [28, 29]. Earlier studies have shown that the results of unweighted and weighted indices can differ sharply [4, 25], which is consistent with our findings. Extreme prices can skew the results of unweighted indices, so these indices are generally considered less reliable than weighted ones for comparing drug prices [25].

There is no consensus on which weighting method is most appropriate for comparing drug prices, as each has advantages and disadvantages [12, 25]. Academic and government studies have variously calculated unweighted [9, 10], Fisher [11], Paasche [4, 25], and Laspeyres indices [4, 17, 25, 30], often using different units of volume and/or base countries. The likely reason why Paasche results are usually lower than Laspeyres results, a finding which has been reported in previous drug price indices [4, 25], is that patients tend to consume more of the drugs that are cheaper in their countries. Therefore, when prices are weighted by local consumption, the indices show lower average prices — relative to the base country — than when prices are weighted by consumption in the base country.

The choice of unit of volume can influence the results if there are large, systematic differences between countries in the average strength per dose [25]. For example, previous studies have found that price-index results for Japan vary significantly depending on whether number of doses or grams of active ingredient serve as the unit of volume [3, 4, 17, 18, 25]. The authors of those studies attributed this finding to the tendency of Japanese clinicians to prescribe higher quantities of lower-strength products.

Despite such methodological challenges, it is still possible to glean useful information from price indices. In particular, it is important to look for consistency across indices. As an example, our results indicate that Denmark and Sweden had the lowest ex-manufacturer prices in nearly all weighted indices, regardless of whether Germany or France served as the base country. This strongly suggests that generic drugs were cheaper in Denmark and Sweden in 2013 than in the other five countries. By contrast, the French and Italian ex-manufacturer prices were among the highest in all weighted indices. Ideally, the results of price indices should be interpreted alongside other quantitative and qualitative data about the impact of individual policies on drug prices. On their own, price indices do not provide causal evidence on the effects of pricing and reimbursement rules, generic substitution laws, and other factors on the prices of generic drugs.

The findings in this study raise questions which merit further research. Both Sweden and Denmark operate tender-like systems for generic drugs,^7^ which may account for the low prices observed in each country [31, 32]. Tendering refers to the bulk purchase of generic drugs from the manufacturers that offer the lowest prices [33]. More work is needed to understand the impact of tendering on drug prices, and whether any observed price reductions can be sustained over time. There is concern that relying exclusively on tendering to procure generic drugs could create product shortages, drive generic drug firms out of business, and lead to higher generic drug prices over time [33]. There is little evidence, however, on the long-term effects of tendering.

It is also important to examine why there are large differences in the prices of drugs in various therapeutic areas, both within and between countries. Such variation may, in part, reflect market factors. For example, the marketing exclusivity for a drug can expire at different times across high-income countries depending on when the drug was approved in each jurisdiction. Also, some studies have observed an inverse relationship between the number of competitors in the market and generic drug prices [34, 35]. The speed of generic entry, in turn, has been found to be correlated with how much brand-name firms record in revenue in the years leading up to patent expiry [36, 37]. In other words, generic firms tend to prioritize more lucrative drug markets.

### Limitations

This study has limitations, most of which are inherent to drug price indices.

First, the data did not account for confidential discounts, which can be as high as 50% for some generic drugs in certain countries [38]. All list prices may, therefore, not have corresponded to the actual prices paid. However, if profits from discounts accrue to wholesalers or pharmacists, then list prices are more important to payers.

Second, Paasche and Laspeyres indices are underpinned by assumptions about the relationship between generic drug prices and usage which may not always hold. Specifically, the results of Laspeyres indices are valid if demand for prescription medicines is price inelastic. While empirical findings contradict this assumption [39, 40], the Paasche index instead assumes that the consumption pattern in the base country would look exactly like that of the comparator country if both had the same prices. The latter assumption might be less likely to hold true, since there are differences between countries in standards of care, disease prevalence rates, prescription drug coverage, and patient preferences — all of which can affect demand [25].

Third, by restricting the analysis to a common sample of drugs, we reduced the sample size. In some previous price indices for patented drugs, researchers instead conducted a series of comparisons between the base country and one other country at a time, looking at the drugs available in both countries. Such comparisons, which are called bilateral analyses, maximize the sample size for each country pair. We chose to instead calculate what are known as multilateral indices, which compare the prices of a sample of drugs available in all study countries. Multilateral indices provide information on how prices compare across all the countries rather than just between each pair. While a common sample might over-represent older, internationally available products [25], this is less of a concern when comparing generic drug prices. However, it is important to note that two countries with identical prices could show up as having differing price levels in a Paasche index if consumption patterns differ. Thus, multilateral price comparisons using Paasche indices should be interpreted with caution.

Fourth, we used common units of volume to aggregate data across formulations of active ingredients [25]. In using prices per dose, however, we assumed that a dose of a drug provides the same therapeutic benefit to any patient, regardless of strength-form combination. By contrast, prices per gram of active ingredient are sensitive to the selection of drugs, given that drug strengths often vary considerably between drugs [11]. The price per defined daily dose is an alternative metric. A defined daily dose is the “assumed average maintenance dose per day for a drug used for its main indication in adults.” [41] We could not identify this dose for each drug in our dataset, as we did not have information about drug indications. However, defined daily doses are not always of equal therapeutic value to all patients, and they may not accurately reflect consumption patterns [25]. For example, a defined daily dose is not adjusted for differences in the duration of treatment. They are, therefore, not necessarily a better unit of comparison than doses or grams of active ingredient [25, 41]. Also, because defined daily doses are specified in terms of grams of active ingredient per day, indices based on defined daily doses and indices based on grams should generate similar findings if the average number of treatment days are fairly consistent across countries for most drugs [4].

Fifth, the drugs were listed by active ingredient, and no information was available on the indications for which the drugs were prescribed. However, a prior study found that the results of price indices were “virtually unchanged” when products were defined by active ingredient instead of active ingredient plus indication [25].

Lastly, we had to exclude 6.7% of drugs (213/3156) due to missing volume data.

## Conclusions

Generic drug policy is an important topic given rising drug expenditures and concerns about the financial sustainability of many health-care systems. More research is needed to better understand the causes of variation in the prices of generic drugs across countries. This will help to identify which measures are most effective at reducing prices. Our findings suggest that some countries should focus on containing the distribution costs for generic drugs.

There are a number of methodological issues that can arise when trying to compare drug prices internationally. Drugs often differ across countries in terms of names, pack sizes, formulations, strengths, and manufacturers. They can also vary in terms of whether they are sold over-the-counter or through prescriptions, and whether they are sold in hospital or retail pharmacies. There is a trade-off between matching all of these factors — which produces more accurate price comparisons of individual products — and the sample size.

Once a sample of drugs has been chosen, there are various ways of calculating price indices to aggregate the data, each with its own advantages and disadvantages, as discussed in this paper. There is no gold standard for comparing drug prices. Our results showed that such comparisons are highly sensitive to the choice of method — for example, Laspeyres versus Paasche indices — which is consistent with the findings of earlier studies of patented drugs.

Overall, price indices are a useful statistical approach for comparing drug prices across countries, but policymakers and researchers should interpret price indices with caution given their limitations.

## Additional file

Additional file 1: Appendix 1. List of the 200 most-prescribed off-patent active ingredients in Europe in 2013 (anatomical main group in parentheses). **Appendix 2**. Ex-manufacturer and retail prices with France as the base country (2013). **Appendix 3**. Ex-manufacturer and retail prices based on PPP adjustments with Germany as the base country (2013). **Appendix 4**. Ex-manufacturer and retail prices of cardiovascular and nervous system drugs with Germany as the base country (2013). (DOCX 58 kb)

## Abbreviations

ATC/DDD: Anatomical therapeutic chemical classification system with defined daily doses
OECD: Organization for Economic Co-operation and Development
PPP: Purchasing power parity
UK: United Kingdom
US: United States

## Footnotes

1 The dataset excluded generic drugs sold in hospital pharmacies, off-patent originator drugs, parallel-traded products, and off-patent biological drugs.

2 IMS Health refers to doses as “standard units.”

3 These values were calculated by multiplying the number of packs sold of each product by the corresponding prices on a quarterly basis. For these calculations, IMS Health relied on the latest prices in each quarter from validated sources, such as government price lists and wholesaler invoices, excluding value-added taxes.

4 If sales of either <1,000 doses or < €1,000 were recorded in a country for a drug, we decided *a priori* to exclude the sales figures for that country, as was done in previous studies. Those values may reflect data-entry errors or inconsistencies in reporting across countries.

5 For the common sample of 110 active ingredients, the average number of grams of active ingredient per dose ranged from 0.09 grams in Sweden to 0.19 grams in Spain.

6 For ease of comparison to the other results, all prices are expressed in relation to those in Germany (index value = 100).

7 National government authorities in Denmark and Sweden operate tender-like systems: the relevant authority in each country asks drug makers to offer their best prices, and, in most cases, the cheapest products are the only ones which public payers will reimburse. This bidding process is repeated every two and four weeks in Denmark and Sweden, respectively. Payers in Germany and Spain also tender for generic drugs, but the tender results were kept confidential in 2013. The data, therefore, did not reflect tendering outcomes in either country.
