# Supplementary material for: A comparison of generic drug prices in seven European countries: a methodological analysis
Source: BMC Health Serv Res. 2017 Mar 31;17:242. doi: 10.1186/s12913-017-2184-5 (PMC5374620; doi:10.1186/s12913-017-2184-5)
Supplement: Additional file 1: Appendix 1. — List of the 200 most-prescribed off-patent active ingredients in Europe in 2013 (anatomical main group in parentheses). Appendix 2. Ex-manufacturer and retail prices with France as the base country (2013). Appendix 3. Ex-manufacturer and retail prices based on PPP adjustments with Germany as the base country (2013). Appendix 4. Ex-manufacturer and retail prices of cardiovascular and nervous system drugs with Germany as the base country (2013). (DOCX 58 kb) [file 12913_2017_2184_MOESM1_ESM.docx]

Appendix 1. List of the 200 most-prescribed off-patent active ingredients in Europe in 2013 (anatomical main group in parentheses) ^a^

| 1. Acetylcysteine (R/S/V) | 1. Acetylsalicylic acid (A/B/N) |
| --- | --- |
| 1. **Aciclovir (D/J/S)** | 1. **Alendronic acid (M)** |
| 1. **Alfuzosin (G)** | 1. **Allopurinol (M)** |
| 1. **Alprazolam (N)** | 1. Alprostadil (C/G) |
| 1. Amiodarone (C) | 1. Amisulpride (N) |
| 1. Amitriptyline (N) | 1. **Amlodipine (C)** |
| 1. Amlodipine / perindopril (C) | 1. **Amoxicillin (J)** |
| 1. **Amoxicillin / clavulanic acid (J)** | 1. **Anastrozole (L)** |
| 1. Apomorphine (G/N) | 1. **Atenolol (C)** |
| 1. **Atorvastatin (C)** | 1. Azathioprine (L) |
| 1. **Azithromycin (J/S)** | 1. **Beclometasone (A/D/R)** |
| 1. Betahistine (N) | 1. Betamethasone (A/C/D/H/R/S) |
| 1. **Bicalutamide (L)** | 1. **Bisoprolol (C)** |
| 1. Bisoprolol / hydrochlorothiazide (C) | 1. Bromazepam (N) |
| 1. **Budesonide (A/D/R)** | 1. Buprenorphine (N) |
| 1. **Candesartan cilexetil (C)** | 1. **Candesartan cilexetil / hydrochlorothiazide (C)** |
| 1. **Carbamazepine (N)** | 1. Carbidopa / levodopa (N) |
| 1. **Carvedilol (C)** | 1. Cefpodoxime proxetil (J) |
| 1. Ceftriaxone (J) | 1. Cefuroxime axetil (J/S) |
| 1. Cetirizine (R) | 1. Ciclosporin (L/S) |
| 1. **Ciprofloxacin (J/S)** | 1. **Citalopram (N)** |
| 1. Clarithromycin (J) | 1. **Clindamycin (D/G/J)** |
| 1. **Clopidogrel (B)** | 1. **Clozapine (N)** |
| 1. Codeine (N/R) | 1. Codeine / paracetamol (N) |
| 1. **Cyproterone ethinylestradiol (G)** | 1. **Desloratadine (R)** |
| 1. Desmopressin (H) | 1. Desogestrel / ethinylestradiol (G) |
| 1. **Dexamethasone (A/C/D/H/R/S)** | 1. Diazepam (N) |
| 1. **Diclofenac (D/M/S)** | 1. Dienogest / ethinylestradiol (G) |
| 1. Diltiazem (C) | 1. Docetaxel (L) |
| 1. Domperidone (A) | 1. **Donepezil (N)** |
| 1. Doxazosin (C) | 1. **Doxycycline (A/J)** |
| 1. Drospirenone / ethinylestradiol (G) | 1. Ebastine (R) |
| 1. **Enalapril (C)** | 1. **Enalapril / hydrochlorothiazide (C)** |
| 1. **Erythromycin (D/J/S)** | 1. Escitalopram (N) |
| 1. **Esomeprazole (A)** | 1. **Estradiol (G)** |
| 1. Estradiol / norethisterone (G) | 1. Ethinylestradiol / gestodene (G) |
| 1. **Ethinylestradiol / levonorgestrel (G)** | 1. **Felodipine (C)** |
| 1. Fenofibrate (C) | 1. **Fentanyl (N)** |
| 1. **Finasteride (D/G)** | 1. Flucloxacillin (J) |
| 1. **Fluconazole (D/J)** | 1. **Fluoxetine (N)** |
| 1. Fluticasone / salmeterol (R) | 1. Fluvastatin (C) |
| 1. **Formoterol (R)** | 1. **Furosemide (C)** |
| 1. **Gabapentin (N)** | 1. **Galantamine (N)** |
| 1. Gliclazide (A) | 1. **Glimepiride (A)** |
| 1. **Hyaluronic acid (D/M/R/S)** | 1. Hydrochlorothiazide (C) |
| 1. **Hydrochlorothiazide / lisinopril** **(C)** | 1. **Hydrochlorothiazide / losartan** **(C)** |
| 1. Hydrochlorothiazide / ramipril (C) | 1. **Hydrochlorothiazide / valsartan** **(C)** |
| 1. **Hydrocortisone (A/C/D/H/S)** | 1. Hydromorphone (N) |
| 1. **Ibandronic acid (M)** | 1. **Ibuprofen (C/G/M)** |
| 1. Indapamide (C) | 1. Indapamide / perindopril (C) |
| 1. Iodine / levothyroxine sodium (H) | 1. **Ipratropium bromide (R)** |
| 1. **Irbesartan (C)** | 1. Irinotecan (L) |
| 1. Isosorbide mononitrate (C) | 1. Isotretinoin (D) |
| 1. Ketoprofen (M) | 1. **Lamotrigine (N)** |
| 1. **Lansoprazole (A)** | 1. **Latanoprost (S)** |
| 1. Leflunomide (L) | 1. **Lercanidipine (C)** |
| 1. **Letrozole (L)** | 1. Leuprorelin (L) |
| 1. **Levetiracetam (N)** | 1. Levocetirizine (R) |
| 1. Levofloxacin (J/S) | 1. **Levothyroxine sodium (H)** |
| 1. **Lidocaine (C/D/N/R/S)** | 1. **Lisinopril (C)** |
| 1. **Lorazepam (N)** | 1. Lormetazepam (N) |
| 1. **Losartan (C)** | 1. **Memantine (N)** |
| 1. Mesalazine (A) | 1. Metamizole sodium (N) |
| 1. **Metformin (A)** | 1. Methadone (N) |
| 1. **Methotrexate (L)** | 1. Methylphenidate (N) |
| 1. **Metoprolol (C)** | 1. **Metronidazole (A/D/G/J/P)** |
| 1. **Mirtazapine (N)** | 1. Molsidomine (C) |
| 1. **Montelukast (R)** | 1. **Morphine (N)** |
| 1. Moxonidine (C) | 1. Naloxone / tilidine (N/V) |
| 1. **Naproxen (G/M)** | 1. Nebivolol (C) |
| 1. Nifedipine (C) | 1. **Nitroglycerin (C)** |
| 1. Ofloxacin (J/S) | 1. **Olanzapine (N)** |
| 1. **Omeprazole (A)** | 1. **Ondansetron (A)** |
| 1. Oxaliplatin (L) | 1. **Oxycodone (N)** |
| 1. Paclitaxel (L) | 1. **Pantoprazole (A)** |
| 1. Paracetamol (N) | 1. Paracetamol / tramadol (N) |
| 1. **Paroxetine (N)** | 1. Penicillin (J/S) |
| 1. Perindopril (C) | 1. Phenytoin (N) |
| 1. Piracetam (N) | 1. **Pramipexole (N)** |
| 1. **Pravastatin (C)** | 1. **Prednisolone (A/C/D/H/R/S)** |
| 1. **Prednisone (A/H)** | 1. **Progesterone (G)** |
| 1. Propranolol (C) | 1. Quetiapine (N) |
| 1. Rabeprazole (A) | 1. **Ramipril (C)** |
| 1. Ranitidine (A) | 1. **Repaglinide (A)** |
| 1. Rilmenidine (C) | 1. **Risedronic acid (M)** |
| 1. **Risperidone (N)** | 1. **Ropinirole (N)** |
| 1. Rosuvastatin (C) | 1. **Salbutamol (R)** |
| 1. **Sertraline (N)** | 1. **Sildenafil (G)** |
| 1. **Simvastatin (C)** | 1. **Spironolactone (C)** |
| 1. **Sumatriptan (N)** | 1. Tamsulosin (G) |
| 1. Temazepam (N) | 1. Temozolomide (L) |
| 1. **Terbinafine (D)** | 1. **Testosterone (G)** |
| 1. **Timolol (C/S)** | 1. Tolterodine (G) |
| 1. **Topiramate (N)** | 1. Torasemide (C) |
| 1. **Tramadol (N)** | 1. Trazodone (N) |
| 1. Trimebutine (A) | 1. Trimetazidine (C) |
| 1. **Valaciclovir (J)** | 1. **Valproic acid (N)** |
| 1. **Valsartan (C)** | 1. **Venlafaxine (N)** |
| 1. Verapamil (C) | 1. Warfarin (B) |
| 1. **Zolpidem (N)** | 1. **Zopiclone (N)** |

^a^ The data included over-the-counter products, such as ibuprofen and paracetamol, if these were prescribed by a licensed health-care practitioner. The active ingredients listed in bold were available in at least one form-strength combination in each country. These 110 ingredients formed the common sample.

***Source***: IMS Health 2013 (Pricing Insights database); anatomical main groups from the WHOCC ATC/DDD Index (2015).

The breakdown of active ingredients in the common sample (n = 110) by anatomical main group.

| **Code** | **Anatomical main group** | **Count** |
| --- | --- | --- |
| **A** | Alimentary tract and metabolism | 8 |
| **B** | Blood and blood-forming organs | 1 |
| **C** | Cardiovascular system | 25 |
| **D** | Dermatologicals | 0^a^ |
| **G** | Genito-urinary system and sex hormones | 7 |
| **H** | Systemic hormonal preparations (excl. sex hormones and insulins) | 1 |
| **J** | Anti-infectives for systemic use | 3 |
| **L** | Antineoplastic and immunomodulating agents | 4 |
| **M** | Musculo-skeletal system | 4 |
| **N** | Nervous system | 29 |
| **P** | Antiparasitic products, insecticides and repellants | 0^a^ |
| **R** | Respiratory system | 5 |
| **S** | Sensory organs | 1 |
| **V** | Various | 0 |
| - | Belong to multiple anatomical main groups | 22 |

^a^ All D and P medicines belonged to multiple groups

***Source***: WHOCC ATC/DDD Index (2015).

**Appendix 2**. Ex-manufacturer and retail prices with France as the base country (2013)

|  | **Belgium** | **Denmark** | **France** | **Germany** | **Italy** | **Spain** | **Sweden** |
| --- | --- | --- | --- | --- | --- | --- | --- |
| **Ex-manufacturer prices** | | | | | | | |
| Unweighted-D | 78.59 | 48.36 | 100.00 | 100.69 | 110.61 | 137.81 | 65.81 |
| Unweighted-G | 181.48 | 86.56 | 100.00 | 157.19 | 79.49 | 99.17 | 90.04 |
| Laspeyres-D | 106.44 | 80.64 | 100.00 | 114.84 | 138.80 | 117.96 | 77.23 |
| Laspeyres-G | 99.42 | 77.05 | 100.00 | 114.22 | 127.40 | 94.44 | 85.43 |
| Paasche-D | 98.02 | 29.00 | 100.00 | 69.22 | 91.58 | 68.71 | 51.07 |
| Paasche-G | 79.74 | 28.07 | 100.00 | 60.64 | 79.08 | 62.68 | 50.09 |
| Fisher-D | 102.14 | 48.36 | 100.00 | 89.16 | 112.74 | 90.03 | 62.80 |
| Fisher-G | 89.04 | 46.50 | 100.00 | 83.22 | 100.37 | 76.94 | 65.41 |
| **Retail prices** | | | | | | | |
| Unweighted-D | 72.14 | 49.77 | 100.00 | 101.65 | 119.51 | 131.74 | 53.78 |
| Unweighted-G | 182.51 | 102.33 | 100.00 | 175.35 | 99.89 | 110.03 | 83.27 |
| Laspeyres-D | 108.28 | 87.73 | 100.00 | 160.86 | 147.25 | 111.29 | 78.87 |
| Laspeyres-G | 101.04 | 84.89 | 100.00 | 158.94 | 134.96 | 89.03 | 86.43 |
| Paasche-D | 98.30 | 36.75 | 100.00 | 103.03 | 99.04 | 63.62 | 54.21 |
| Paasche-G | 79.61 | 35.29 | 100.00 | 87.07 | 85.97 | 57.72 | 52.45 |
| Fisher-D | 103.17 | 56.78 | 100.00 | 128.74 | 120.76 | 84.15 | 65.39 |
| Fisher-G | 89.69 | 54.73 | 100.00 | 117.64 | 107.71 | 71.69 | 67.33 |

D, doses; G, grams of active ingredient

***Source***: IMS Health 2013 (Pricing Insights database).

**Appendix 3**. Ex-manufacturer and retail prices based on PPP adjustments with Germany as the base country (2013)

|  | **Belgium** | **Denmark** | **France** | **Germany** | **Italy** | **Spain** | **Sweden** |
| --- | --- | --- | --- | --- | --- | --- | --- |
| **Ex-manufacturer** | | | | | | | |
| Unweighted-D | 74.39 | 37.15 | 94.28 | 100.00 | 114.45 | 157.94 | 51.18 |
| Unweighted-G | 110.04 | 42.60 | 60.39 | 100.00 | 52.69 | 72.81 | 44.86 |
| Laspeyres-D | 130.26 | 47.27 | 137.13 | 100.00 | 163.24 | 155.18 | 67.11 |
| Laspeyres-G | 120.95 | 53.09 | 156.56 | 100.00 | 160.67 | 143.40 | 79.55 |
| Paasche-D | 103.66 | 26.60 | 82.66 | 100.00 | 110.02 | 90.38 | 44.24 |
| Paasche-G | 92.63 | 30.91 | 83.11 | 100.00 | 66.56 | 75.32 | 52.80 |
| Fisher-D | 116.20 | 35.46 | 106.47 | 100.00 | 134.01 | 118.43 | 54.49 |
| Fisher-G | 105.85 | 40.51 | 114.07 | 100.00 | 103.41 | 103.92 | 64.81 |
| **Retail** | | | | | | | |
| Unweighted-D | 67.65 | 37.87 | 93.39 | 100.00 | 122.50 | 149.57 | 41.43 |
| Unweighted-G | 99.21 | 45.15 | 54.14 | 100.00 | 59.36 | 72.41 | 37.19 |
| Laspeyres-D | 87.91 | 37.35 | 92.14 | 100.00 | 119.48 | 99.26 | 46.29 |
| Laspeyres-G | 83.19 | 42.49 | 109.03 | 100.00 | 116.99 | 91.42 | 54.71 |
| Paasche-D | 67.15 | 25.14 | 59.01 | 100.00 | 79.48 | 53.58 | 34.46 |
| Paasche-G | 60.99 | 28.84 | 59.73 | 100.00 | 49.73 | 46.79 | 41.26 |
| Fisher-D | 76.83 | 30.64 | 73.74 | 100.00 | 97.45 | 72.93 | 39.94 |
| Fisher-G | 71.23 | 35.01 | 80.70 | 100.00 | 76.28 | 65.41 | 47.51 |

D, doses; G, grams of active ingredient

***Source***: IMS Health 2013 (Pricing Insights database).

**Appendix 4**. Ex-manufacturer and retail prices of cardiovascular and nervous system drugs with Germany as the base country (2013)

Cardiovascular system active ingredient (n = 25)

|  | **Belgium** | **Denmark** | **France** | **Germany** | **Italy** | **Spain** | **Sweden** |
| --- | --- | --- | --- | --- | --- | --- | --- |
| **Ex-manufacturer** | | | | | | | |
| Unweighted-D | 132.27 | 93.24 | 138.78 | 100.00 | 108.37 | 131.40 | 61.37 |
| Unweighted-G | 36.83 | 69.01 | 45.56 | 100.00 | 31.82 | 36.26 | 33.94 |
| Laspeyres-D | 218.08 | 67.45 | 235.22 | 100.00 | 155.92 | 178.09 | 96.41 |
| Laspeyres-G | 193.33 | 64.03 | 262.42 | 100.00 | 169.33 | 176.21 | 107.84 |
| Paasche-D | 186.92 | 48.96 | 161.27 | 100.00 | 128.38 | 130.94 | 64.06 |
| Paasche-G | 106.46 | 46.44 | 117.94 | 100.00 | 33.18 | 58.58 | 72.16 |
| Fisher-D | 201.90 | 57.47 | 194.77 | 100.00 | 141.48 | 152.70 | 78.59 |
| Fisher-G | 143.46 | 54.53 | 175.93 | 100.00 | 74.95 | 101.59 | 88.21 |
| **Retail** | | | | | | | |
| Unweighted-D | 90.57 | 69.55 | 99.80 | 100.00 | 86.25 | 88.17 | 48.71 |
| Unweighted-G | 24.12 | 44.57 | 30.43 | 100.00 | 23.90 | 23.00 | 25.40 |
| Laspeyres-D | 113.15 | 46.28 | 128.59 | 100.00 | 91.25 | 86.76 | 61.19 |
| Laspeyres-G | 99.94 | 44.82 | 143.99 | 100.00 | 99.15 | 85.93 | 68.32 |
| Paasche-D | 101.70 | 37.28 | 104.37 | 100.00 | 82.95 | 74.95 | 47.63 |
| Paasche-G | 63.24 | 36.23 | 77.73 | 100.00 | 24.45 | 36.06 | 53.52 |
| Fisher-D | 107.27 | 41.53 | 115.85 | 100.00 | 87.00 | 80.64 | 53.99 |
| Fisher-G | 79.50 | 40.30 | 105.79 | 100.00 | 49.23 | 55.67 | 60.47 |

D, doses; G, grams of active ingredient

***Source***: IMS Health 2013 (Pricing Insights database).

Nervous system active ingredients (n = 29)

|  | **Belgium** | **Denmark** | **France** | **Germany** | **Italy** | **Spain** | **Sweden** |
| --- | --- | --- | --- | --- | --- | --- | --- |
| **Ex-manufacturer** | | | | | | | |
| Unweighted-D | 74.20 | 28.15 | 87.51 | 100.00 | 84.09 | 91.41 | 47.57 |
| Unweighted-G | 137.93 | 45.23 | 56.84 | 100.00 | 43.70 | 55.74 | 20.52 |
| Laspeyres-D | 94.62 | 32.29 | 100.38 | 100.00 | 98.14 | 89.11 | 58.15 |
| Laspeyres-G | 103.92 | 36.06 | 97.08 | 100.00 | 80.77 | 82.69 | 64.80 |
| Paasche-D | 102.02 | 27.70 | 93.40 | 100.00 | 118.60 | 87.31 | 51.84 |
| Paasche-G | 113.27 | 32.57 | 96.33 | 100.00 | 111.16 | 75.89 | 55.55 |
| Fisher-D | 98.25 | 29.91 | 96.83 | 100.00 | 107.89 | 88.21 | 54.90 |
| Fisher-G | 108.49 | 34.27 | 96.70 | 100.00 | 94.75 | 79.21 | 60.00 |
| **Retail** | | | | | | | |
| Unweighted-D | 65.19 | 31.71 | 77.06 | 100.00 | 91.51 | 85.08 | 40.67 |
| Unweighted-G | 124.59 | 50.17 | 50.38 | 100.00 | 50.17 | 55.89 | 18.27 |
| Laspeyres-D | 78.43 | 34.25 | 84.33 | 100.00 | 99.31 | 77.62 | 47.36 |
| Laspeyres-G | 86.15 | 37.94 | 83.98 | 100.00 | 82.60 | 72.55 | 53.61 |
| Paasche-D | 65.12 | 29.90 | 60.59 | 100.00 | 90.42 | 58.44 | 41.42 |
| Paasche-G | 64.49 | 33.97 | 66.05 | 100.00 | 82.54 | 53.66 | 45.16 |
| Fisher-D | 71.47 | 32.00 | 71.48 | 100.00 | 94.76 | 67.35 | 44.29 |
| Fisher-G | 74.53 | 35.90 | 74.48 | 100.00 | 82.57 | 62.39 | 49.20 |

D, doses; G, grams of active ingredient

***Source***: IMS Health 2013 (Pricing Insights database).
